# Atypical Presentations of Foix-Chavany-Marie Syndrome (FCMS) in Stroke

**DOI:** 10.7759/cureus.38030

**Published:** 2023-04-23

**Authors:** Vivek Sanker, Aariya Srinivasan, Mohamed Emara, Preethi Jagannath, Robert Mathew

**Affiliations:** 1 General Surgery, Noorul Islam Institute of Medical Science (NIMS), Trivandrum, IND; 2 Internal Medicine, Saveetha Medical College and Hospital, Tamil Nadu, IND; 3 College of Medicine, University of Sharjah, Sharjah, ARE; 4 Medicine, MS Ramaiah Medical College, Bangalore, IND; 5 Neurology, Sree Mookambika Institute of Medical Science, Trivandrum, IND

**Keywords:** brain infarct, hypertension, anarthria, acute hemiplegia, stroke

## Abstract

Foix-Chavany-Marie syndrome (FCMS) presents with anarthria and bilateral (B/L) central facio-linguo-velo-pharyngo-masticatory paralysis with “autonomic voluntary dissociation.” The most common cause of FCMS is cerebrovascular disease, while rarer causes include central nervous system infection, developmental disorders, epilepsy, and neurodegenerative disorders. Even though this syndrome is also referred to as (B/L) anterior operculum syndrome, patients with lesion in sites other than (B/L) opercular regions also can develop the syndrome. In this article we describe two such atypical cases. *Case 1*: A 66-year-old man with diabetes and hypertension who is a smoker had right-sided hemiplegia one year back developed the syndrome acutely two days before admission. CT brain showed left perisylvian infarct and right internal capsule anterior limb infarct. *Case 2*: A 48-year-old gentleman, who is a diabetic and hypertensive had right-sided hemiplegia one year back and developed the syndrome acutely two days before admission. CT brain showed (B/L) infarcts in the posterior limb of the internal capsule. Both patients had bifacial, lingual, and pharyngolaryngeal palsy thereby confirming the diagnosis of FCMS. None of them had the classical (B/L) opercular lesions on imaging and one patient did not even have a unilateral opercular lesion. Contrary to the common teaching, (B/L) opercular lesions are not always necessary to produce FCMS and can occur even without opercular lesions at all.

## Introduction

Foix-Chavany-Marie syndrome (FCMS) or anterior bilateral (B/L) anterior opercular syndrome is a rare type of pseudobulbar palsy [1]. The B/L anterior opercular syndrome was first reported by Magnus in 1837 and described by Foix et al. in the 1920s [2-3]. FCMS is commonly caused by B/L lesions of the anterior operculum in which motor fibers of cranial nerves V, VII, IX, X, and XII are present [1]. FCMS presents with a characteristic loss of voluntary control over the pharyngeal, lingual, facial, and masticatory muscles with preserved reflexive and autonomic functions [4]. Preserved reflexes and autonomic function with paralysis of voluntary movement is termed autonomic-voluntary movement dissociation [5]. Patients with autonomic-voluntary movement dissociation are unable to voluntarily open their mouths, show their teeth, blow their cheeks, or speak, however, their emotional and automatic functions are intact [5]. They can yawn, laugh, and cry. The most common cause of FCMS is a cerebrovascular disease specifically ischemic stroke [6]. Other etiologies described in the literature include epilepsy, infections, neoplasia, vasculitis, insular glioma surgery, cortical dysplasia, traumatic brain injury, and degenerative diseases [4-6]. Although FCMS is usually caused by B/L opercular cerebrovascular disease leading to ischemic strokes, unilateral lesions causing FCMS has been described before [6]. In this article, we describe two such atypical cases causing FCMS. 

## Case presentation

Case 1

A 66-year-old male presented to the emergency department with acute onset of right-sided weakness which started 3 months back. He also complained of dysphagia and anarthria along with weakness. His past medical history was significant for hypertension and type 2 diabetes mellitus from 15 years and had taken cilnidipine 10 mg and sitagliptin 100 mg respectively for the same irregularly. He had a stroke one year ago with mild residual deficit persisting as left-sided hemiparesis. He was not taking any antiplatelets or anti-coagulants. Additionally, he also has a history of chronic smoking (20 pack years).

**Figure 1:**
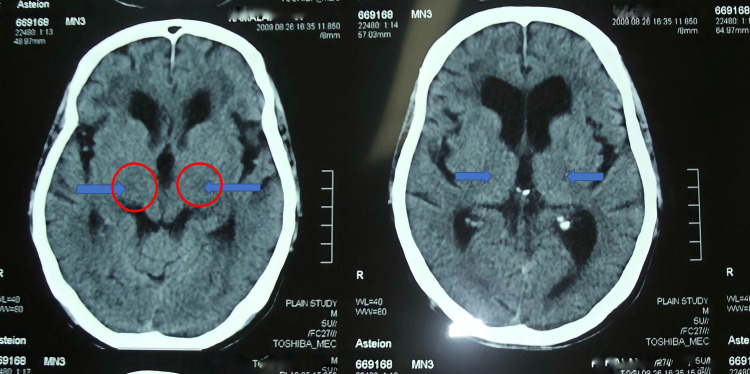
CT brain delineated an acute left peri-sylvian infarct and a chronic right internal capsular anterior limb infarct (shown in blue arrow and red circles).

On general examination, the patient had a normal heart rate, respiratory rate, oxygen saturation, and temperature. On physical examination, he appeared withdrawn from his surroundings. He was unable to speak. He did not show receptive aphasia. He answered various questions appropriately by writing them in a notebook. On admission, his blood pressure was 140/90 mmHg. On neurological examination, he was anarthrous. He had weakness of the lower face associated with difficulty in smiling, puffing out his cheeks, protruding his tongue, or moving his tongue sideways. Deviation of the tongue was not noticed. Tongue movements were slow and showed fasciculations. The range of motion in the jaw showed a marked reduction. His muscle strength was 2/5 in the right upper and lower limbs and 4/5 in the left upper and lower limbs. His muscle tone was spastic in all extremities. Deep tendon reflexes were exaggerated. He showed an extensor plantar reflex on the right. Additionally, palatal movements were bilaterally absent, and palatal reflexes were exaggerated in accordance with a pseudobulbar syndrome. Cerebellar testing yielded normal results on examination. CT brain delineated a left perisylvian infarct and a right internal capsular anterior limb infarct (Figure 1). MRI was not done due to affordability issues. He was initiated on Aspirin, Atorvastatin, antihypertensive, and anti-diabetes medications. By 2 weeks of illness, at the time of discharge, his limb power had started to improve but he was on nasogastric tube feeding. He was reviewed after 1 month. His limb power had improved significantly but he continued to be anarthrous. He continued to be on nasogastric tube in view of significant dysphagia.

Case 2

A 48-year-old male who was a known case of hypertension and type 2 diabetes (not on regular medication) presented with acute onset dysphagia and anarthria 2 weeks prior to presentation. He suffered from a stroke one year back and had residual deficits of right-sided hemiparesis.

**Figure 2:**
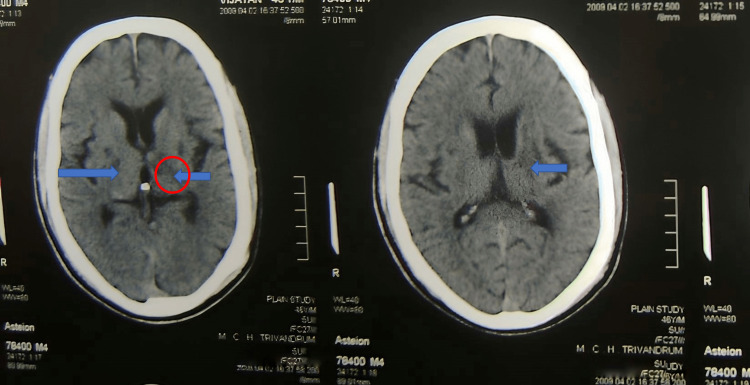
CT brain showing bilateral infarcts in the posterior limb of internal capsule (shown in blue arrow and red circle).

On investigation, the CT brain showed (B/L) infarcts in the posterior limb of the internal capsule (acute ischemic infarct in the posterior limb of the right internal capsule and chronic infarct in the left posterior limb of the internal capsule) (Figure 2). On examination, he was found to be anarthrous. He had dysarthria with pooling of saliva in the throat. He had spasticity with grade 4 power on the right and normal tone with grade 4 power on the left. He was diagnosed as having acute ischemic stroke and put on dual antiplatelets in addition to statin, anti-diabetics, and anti-hypertensive. He had to be on a nasogastric feeding tube. Two weeks after the onset of the illness at the time of discharge he continued to be on nasogastric tube and was anarthrous.

## Discussion

Foix Chavany Marie syndrome also called (B/L) opercular syndrome is a form of pseudo bulbar palsy causing facio-labio-pharyngo-laryngo-glosso-brachial paralysis but with preserved autonomic and reflex movements also known as automatic-voluntary dissociation [7]. Though there are many causes for the syndrome, vascular etiology is found to be the major cause. In both the cases described here, vascular etiology was seen to be the cause. A literature review done by Weller showed five clinical types of FCMS: (a) classical and most common cerebrovascular disease; (b) central nervous system (CNS) infections causing subacute form; (c) developmental form probably caused by neuronal migration disorders; (d) children with epilepsy which is reversible; and (e) rare form by neurodegenerative disorders [8].

Though the actual incidence of the syndrome is not known till now, varied etiologies have been identified and documented in various studies including, perinatal insults, inflammatory demyelinating syndromes, developmental malformations like heterotopias, polymicrogyria, and infective etiology [9]. Classically FCMS is attributed to (B/L) frontal anterior opercular lesions which contain motor fibers from cranial nerves V, VII, IX, X, and XII though (B/L) posterior, unilateral anterior, and unilateral posterior lesions may also occur [10]. Contrary to the common findings, both cases mentioned here presented with bifacial, lingual, and pharyngolaryngeal palsy thereby confirming the diagnosis of FCMS but none of them had the classical (B/L) frontal opercular lesions on imaging.

In the first case mentioned, CT brain showed a left perisylvian infarct and a right internal capsule anterior limb infarct. Though typically only (B/L) opercular lesions can cause this syndrome, any lesions involving combinations of cortical or subcortical areas of the operculum or its connections on both sides of the brain can produce a syndrome similar to this [11]. In a study done by Brandoe et al., one possible hypothesis has been documented which states the existence of an anatomical variant, such that there is predominantly unilateral corticobulbar tract representation [6]. While, the majority of the patients who are diagnosed with FCMS due to (B/L) opercular lesions have a poor prognosis with residual defects in swallowing, chewing, and speaking and often require medical assistance due to the risk of aspiration, patients with unilateral opercular lesions have a relatively good prognosis [4, 10]. While the exact mechanism and pathophysiology/anatomical basis of FCMS is not known, some other studies have hypothesized that the presence of asynchronous contralateral lesions could catalyze the development of FCMS [1, 12-13].

Further, in the second case described, a CT scan of the brain showed (B/L) infarcts in the posterior limb of the internal capsule possibly affecting (B/L) cortico-bulbar tracts, and no opercular lesions were found suggesting that when encountering any patient recovering from stroke with features of FCMS, a diagnosis of FCMS can be made even if there are no classical opercular lesions as this unique syndrome is not only associated with opercular lesions. Kobayashi et al. in a study, described a case of FCMS and crossed aphasia after isolated right corona radiata infarction and a history of left hemispheric infarction [14]. Another study done by Kozak et al. described FCMS that occurred secondary to isolated pontine infarct [15]. In a study done by Kaloostian et al., reversible FCMS was diagnosed in a patient who was treated for hydrocephalus [7].

Many reported cases of FCMS in the literature did not undergo extensive imaging [16]. These cases were most probably reported before the introduction of nuclear scanning [16]. In both cases mentioned, because of financial constraints patients did not undergo further imaging. SPECT scans have previously ruled out unilateral FCMS by providing evidence of hypoperfusion bilaterally [16]. Thus, the existence of unilateral FCMS is still considered controversial [16]. It is hard to associate a specific cortical area within the operculum causing FCMS. Most FCMS cases are due to cerebrovascular disease and thus a broad cortical and subcortical area is affected [5]. Unilateral damage of the subcortical connections of the inferior frontal operculum gyrus with the frontal aslant tract (FAT) and arcuate fasciculus (AF) may produce transient FCMS. Martino et al. described a case of transient FCMS due to iatrogenic damage of the FAT and AF [5]. Tractography reconstruction of FAT and AF revealed the structural substrate of FCMS [5]. In addition, unilateral representation of the motor centers involved in FCMS was excluded by functional MRI [5]. However, contralateral insular-opercular hypometabolism was not excluded [5]. Thus, when encountering a patient recovering from a stroke with FCMS, we should consider the possibility that this syndrome is not only associated with (B/L) opercular lesions but also due to the involvement of opercular fibers in the subcortical regions. 

## Conclusions

A diagnosis of FCMS should be considered in a patient presenting with loss of voluntary control over the pharyngeal, lingual, facial, and masticatory muscles with preserved reflexive and automatic functions, even if it is a relatively rare neurological disorder and in the absence of common (B/L) opercular lesions as its often neglected or missed. Early recognition of the condition is crucial for prompt intervention, rehabilitation, nutritional support, and secondary prevention. The lack of extensive neurological imaging and assessment probably due to financial constraints as described here could be a possible reason for the controversial existence of unilateral FCMS.
